# Case Report: Intraoperative Fascial Traction in Robotic Abdominal Wall Surgery; An Early Experience

**DOI:** 10.3389/jaws.2022.10356

**Published:** 2022-03-28

**Authors:** A. L. A. Bloemendaal

**Affiliations:** Department of Surgery, Reinier de Graaf Gasthuis, Delft, Netherlands

**Keywords:** introperative fascial traction, hernia, robotic surgery, retromuscular mesh repair, robotic abdominal wall repair

## Abstract

Intraoperative fascial traction (IFT) may obviate the use of a posterior component separation/transversus abdominis release (TAR). Robotic abdominal wall surgery leads to a reduction of morbidity in TAR compared to open surgery. The combination of minimally invasive (robotic) abdominal wall surgery with IFT may lead to a further reduction of surgical morbidity.

## Introduction

Abdominal wall hernia repairs (AWR) are amongst the most common surgical procedures (1). In the case of large hernias, midline closure can be impossible without performing a component separation. Posterior component separation by m. Transversus abdominis release (TAR) has become the most utilized technique, showing clinical superiority over anterior component separation (2, 3).

In recent years, minimally invasive techniques for AWR have developed rapidly. One important drawback, however, of the earlier (laparoscopic) minimally invasive surgical (MIS) techniques was the intraperitoneal placement of the mesh, which may lead to mesh-related complications in the long term (4).

An important addition to these MIS techniques is robot-assisted abdominal wall surgery (RAWS) (5). One clear advantage is the relative ease with which mesh can be placed outside the abdominal cavity in the retrorectus or preperitoneal plane. In addition, the performance of a TAR is possible using a robot (6). Both the open and the robotic TAR are gaining popularity. The clinical benefits of robotic TAR over open TAR have been shown in retrospective settings (3, 7). The main benefit is the shorter length of stay and reduction in wound-related complications.

However, TAR has important setbacks and pitfalls in both open and MIS settings.

Firstly, it is a challenging technique to learn and perform, especially in robotic surgery. The lateral abdominal wall can be an anatomical challenge, and patients needing a component separation have often undergone multiple previous abdominal operations. This may lead to more difficulties, such as the absence of intact fascial layers, or scar tissue, hampering the dissection of the anatomical layers. Unintentional damage to the abdominal wall or its innervation/vasculature is an important risk.

A relatively new technique to achieve medialization of the medial edges of the anterior fascia (i.e. hernia closure) is the use of intraoperative fascial traction (IFT). The first report was the use of AWEX (abdominal wall expansion), a relatively crude technique to achieve medialization but one that shows good results (8). Further development of this technique has led to FascioTens Hernia (FascioTens GmbH, Germany), a device specially developed for IFT, which has shown promising results in open surgery of major midline hernia’s (9). In selected cases, the use of FascioTens obviates the use of component separation for midline closure.

The combination of RAWS and IFT could lead to a further clinical improvement, with significantly lower morbidity in large hernia repairs. This report describes the first early steps into this combination of techniques: a proof of principle.

## Surgical Technique

**FIGURE 1:**
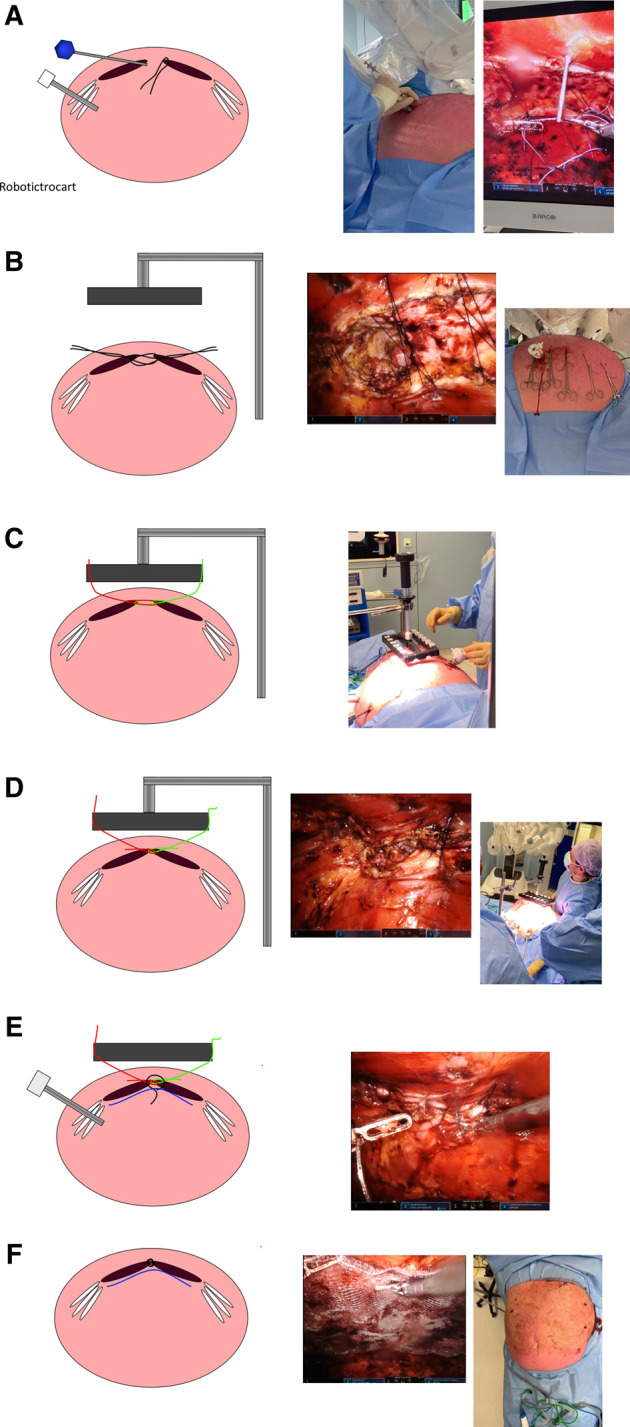
Procedural steps of FascioTens in robotic AWS: **(A)** Placement of vicryl suture in anterior fascia and exteriorisation of suture with placement of endoclosure device through the contralateral anterior fascia. **(B)** FascioTens placed. **(C)** Sutures attached to FascioTens device. **(D)** Gradual increase of tension of the FascioTens until anterior fascia is approximated. **(E)** Once fascia approximated, closure of midline with V-lock 0, placement of mesh. **(F)** Fascia closed, mesh placed; end of procedure.

The operation is performed on a DaVinci Xi robotic platform (Intuïtive Surg, Sunnyvale, CA, United States) using the FascioTens Hernia (FascioTens GmbH, Cologne, Germany). Depiction of the procedural steps in Figure 1.

The patient is placed in a supine position. Administer the following: cefazoline as antibiotic prophylaxis, general anesthesia, and a urinary catheter. It is recommended to have the patient on a continuous muscle-relaxant drip to achieve maximal relaxation.

Perform a sterile exposition of the abdomen. Insufflation of the abdomen is carried out with a veress needle to the left upper quadrant (Palmer’s point) with a pressure of 12–15 mmHg. Place the first robotic trocart with a stump dilator in the left flank. Place additional robotic trocarts as in normal robotic TARUP.

The procedure to be followed is essentially no different from a robotic TARUP (10): an ipsilateral dissection into the retrorectus plane with a development from the plane to the midline. Perform a dissection into the preperitoneal plane for the midline cross-over. Perform a dissection into the contralateral retrorectus plane again, and have the development of this plane go no further than the large (thoracoabdominal) neurovascular bundles. Whilst reducing the hernia, keep in mind that closure of the posterior fascia is necessary for coverage of the mesh. An intact (as possible) hernia sac is beneficial.

Once the plane creation is fully completed, a 12 mm assistance trocart is placed in the area to be covered by the mesh (usually through the contralateral rectus muscle in the upper quadrant).

Preparation of sutures for FascioTens should be as follows: use vicryl 0 or vicryl 1 sutures approximately 60 cm in length. Remember that space is limited in robotic surgery, so the longer the suture the more difficult handling will be. Sutures should be placed through the contralateral anterior fascia in a U-shaped bite (in a craniocaudal direction, parallel to the fascial edge). The needle should be cut off straight away to avoid unnecessary needles in the abdomen. There should be transcutaneous placement of the suture passing device (Endoclose, Medtronic USA). Be aware that the angle should be as horizontal as possible to have horizontal traction instead of ventral traction. The suture passer should be passed through the ipsilateral anterior fascia in order to pull the suture from one fascial edge towards the other. The space between sutures should be approximately 1 cm.

Once the contralateral fascial edge is complete, repeat the steps for the ipsilateral fascial edge. Place small clips onto the exterior sutures.

Undock the robot for placement of the FascioTens device. Desoufflate the abdomen, but leave in the trocarts.

Attach the sutures to the FascioTens device as described by the manufacturer. Slowly increase tension. Due to the “lift” of the abdominal wall, it is possible to watch the approximation of the midline using the robotic camera through a trocart. Once the midline is approximated, release the sutures from the FascioTens device one by one, placing the clip on the skin, to preserve tension. Remove the FascioTens device and redock the robot. Insufflate.

Suture the midline closed with v-lock sutures. It may be necessary to—one-by-one—relax the vicryl IFT-sutures somewhat for a proper view of the anterior fascia, which can be pulled out of sight by the FascioTens tension.

Place the mesh of choice, ensuring it is of an adequate length and width. Remember that the use of an assistance trocart will no longer be possible after mesh placement, so the introduction of sutures needed for the remainder of the procedure could be advised to prevent repeated disconnection of robotic instruments for placement of sutures. Do not fully remove the 12 mm trocart—pull it into the subcutis to prevent the loss of pressure.

Close the posterior fascia with v-lock sutures. Undock the robot and close the skin.

Postoperatively, patients will have an abdominal binder only during mobilization, not during rest, for at least two weeks.

## Results

The FascioTens was used in three cases (all male). The cases were selected due to the expectation of difficult midline closures due to the heavy build of the patient. The first patient had a primary hernia following an emergency laparotomy. The second and third cases presented recurrent midline hernias. The last case had mesh *in situ* from a previous hernia repair.

Patient characteristics and (peri)operative results are shown in Table 1.

**TABLE 1:** Patient characteristics and operative results.

Age	Length/weight/BMI	Hernia width (cm)	Mesh size (L × W)	Operative time (min)	LOS^a^
65	1 m 86/114 kg/33.0	10	25 × 15 cm	255	2 days
47	1 m 72/110 kg/37.1	7	25 × 15 cm	171	3 days
48	2 m 02/169 kg/41.2	7	30 × 15 cm	186	2 days

a Length of stay, including day of operation.

## Discussion

In all three cases, the midline was easily approximated using the FascioTens. Midline closure was without any tension during suturing. The added time of the FascioTens technique decreased over the three cases and was approximately one hour in the third case compared to a standard robotic TARUP. On the other hand, in these selected cases, midline closure may have been troublesome, and a (unilateral) TAR may have been necessary, though this was prevented by the use of IFT. The added time of a unilateral TAR is comparable to the use of the FascioTens, but it comes with an increased risk of morbidity and pain.

Closure of very large abdominal wall hernias robotically can be challenging. Defects over 15 cm wide will often cause a troublesome closure, despite the addition of a TAR and the presence of 15 mmHg intraabdominal pressure, which will, in the author’s own experience, lead to lateral muscle elongation. Even smaller (recurrent) hernias in heavily built patients can be difficult to close robotically. The suture-traction deliverable by the robotic technique is sometimes simply not sufficient; the suture will slip through the needle holder or the suture will break. In these cases, running multiple sutures will often end up in sufficient closure, but it can be a struggle. Delivering simultaneous traction to the entire midline and hernia edges, as performed with IFT, can overcome this difficulty and possibly facilitate closure of defects larger than what is normally possible with the robotic technique, as shown in open surgery. Further research into this possible advantage is needed.

One important issue is the hampering of the surgical assistant in placing the suture passing device between the robotic arms. Good visualization is necessary for the assistant to place the suture passer correctly. Also, coaching from the console surgeon is essential at this stage to ensure that the suture is passed well through the anterior fascia but not too laterally.

An important question that needs answering is whether this technique can obviate the necessity of posterior component separation in selected cases. A TAR is not only useful for the decrease of abdominal wall tension for defect closure but also enables placement of wide meshes up to 30 cm in width. The mesh width in a (robotic) retromuscular (Rives-Stoppa/TARUP) repair can usually not exceed 15 cm. Is this enough to prevent recurrence after tension-less closure of the midline in larger hernias? Further research is needed to answer this question.

In general, there are two possible advantages to the use of IFT in RAWS. The first is the abovementioned avoidance of a component separation for achieving midline closure in a defect too large for normal retrorectus repair. The second possible advantage, which still needs exploring, is the possibility of application of a robotic repair in (previously deemed) too large defects for robotic techniques—for instance a hernia width >15 cm.

In conclusion, the use of IFT is feasible in robotic abdominal repair. Early results are good. Longer follow-up and further cases will show whether smaller mesh in retromuscular repair will be sufficient to prevent recurrence in larger hernias. The possibility of achieving midline closure during minimally invasive surgery for very large defects, by application of IFT, still needs exploring.

## Data Availability

The original contributions presented in the study are included in the article/supplementary material, further inquiries can be directed to the corresponding author.
